# Benign and Malignant Oral Lesion Image Classification Using Fine-Tuned Transfer Learning Techniques

**DOI:** 10.3390/diagnostics13213360

**Published:** 2023-11-01

**Authors:** Md. Monirul Islam, K. M. Rafiqul Alam, Jia Uddin, Imran Ashraf, Md Abdus Samad

**Affiliations:** 1 Department of Software Engineering, Daffodil International University, Daffodil Smart City (DSC), Birulia, Savar, Dhaka 1216, Bangladesh; 2 Department of Statistics, Jahangirnagar University, Dhaka 1342, Bangladesh; 3 AI and Big Data Department, Endicott College, Woosong University, Daejeon 34606, Republic of Korea; 4 Department of Information and Communication Engineering, Yeungnam University, Gyeongsan-si 38541, Republic of Korea

**Keywords:** data-efficient image transformer (DeiT), VGG19, oral lesions, MobileNet, benign, transfer learning, malignant

## Abstract

Oral lesions are a prevalent manifestation of oral disease, and the timely identification of oral lesions is imperative for effective intervention. Fortunately, deep learning algorithms have shown great potential for automated lesion detection. The primary aim of this study was to employ deep learning-based image classification algorithms to identify oral lesions. We used three deep learning models, namely VGG19, DeIT, and MobileNet, to assess the efficacy of various categorization methods. To evaluate the accuracy and reliability of the models, we employed a dataset consisting of oral pictures encompassing two distinct categories: benign and malignant lesions. The experimental findings indicate that VGG19 and MobileNet attained an almost perfect accuracy rate of 100%, while DeIT achieved a slightly lower accuracy rate of 98.73%. The results of this study indicate that deep learning algorithms for picture classification demonstrate a high level of effectiveness in detecting oral lesions by achieving 100% for VGG19 and MobileNet and 98.73% for DeIT. Specifically, the VGG19 and MobileNet models exhibit notable suitability for this particular task.

## 1. Introduction

Oral disease is a major global health concern affecting millions of people worldwide. It was the reason for 15,511 deaths across Bangladesh in 2020. More than 50,000 cases of oral cancer were detected during the same time period [1]. Therefore, oral disease is a substantial problem for us in Bangladesh, as we are in the financial region of a developing country [1]. Tobacco is considered the most common risk factor for oral cancer [2]. Consuming betel nuts has been linked to an increased risk of oral lesions and oral cancer in the Indian subcontinent. According to the World Cancer Research Fund (WCRF) [3], this particular cancer ranks 16th in terms of prevalence, with men being affected at the 11th highest rate and women at the 18th highest rate. The primary stage of oral cancer is oral infection. Moreover, it can be characterized by late diagnosis only. So, if it is possible to detect the type of infection automatically, detecting cancer will not be time-consuming and will also help start treatment at the early stage, reducing the death rate due to oral cancer.

The mucosal tissue or oral area can be altered, which is manifested in several lesions, but comparatively localized overbuilding of oral mucosal tissue is considered reactive rather than neoplastic [4]. The most common oral cavities, infections, or lesions are benign and malignant lesions. Benign lesions, or non-cancerous skin lesions, are less harmful than malignant lesions. Malignant lesions contain cancerous cells and are very harmful to human beings. The improper treatment of malignant lesions can cause death. However, both lesions are the results of tumors. These lesions can be detected by their shape, form, or size. Depending on the weather and geographic position, the characteristics of lesions may vary [5].

Unfortunately, sometimes there is a misunderstanding between cancerous and non-cancerous lesions. Some lesions are not cancerous, depending on their growth, which may occur due to low-grade trauma or a lack of oral hygiene and, in some cases, hormonal reasons; pyrogenic granuloma (PG) is one of the cancerous cases. Similarly, benign lesions are rarely considered non-cancerous lesions, which are the first stage of malignant lesions, and proper treatment is not provided, so the patient must face many difficulties, such as physical problems and sometimes death [4]. Oral pathologies can encompass a wide range of conditions, including both malignant and non-malignant conditions. There are some common malignant and non-malignant oral pathologies associated with various factors, such as oral hygiene, hormonal conditions, trauma, and others. Oral squamous cell carcinoma (OSCC), oral verrucous carcinoma, and mucoepidermoid carcinoma are the common malignant oral cancers [6]. Dental caries, gingivitis, and periodontitis are associated with oral hygiene. Oral contraceptive-associated gingivitis and pregnancy gingivitis occur due to hormonal conditions. Accidents, falls, or physical injuries can lead to various oral injuries, including broken teeth, lip lacerations, or jaw fractures, called oral trauma. Oral thrush (Candidiasis) and herpes labialis (cold sores) are infectious oral conditions. Oral cancer may also be affected by salivary gland disorders, like sialadenitis and Sjogren’s Syndrome [7,8].

Automating the detection of these lesions will reduce costs and allow the disease to be diagnosed earlier. Furthermore, perfect detection will help physicians provide the correct treatment and reduce the suffering of patients, as treatment at the last stage is very costly. Artificial intelligence (AI), machine learning (ML), deep learning (DL), transfer learning (TL), and digital image processing can be used to build an automated system for detecting lesions. There are several ways to automate the diagnosis of oral cancer. AI is a computer science field that uses computers and machines to simulate the human mind’s problem-solving and decision-making skills. ML and DL are subfields of AI, but the two terms have different ways of working. ML learns from previous data without any explicit programs and predicts the outcome accurately. DL works like a human brain structure. There are many data types for classification using AI, ML, and DL. Some of them are text data, image data, structured data, unstructured data, sensor data, social data, and so on. The particular problem at hand, the characteristics of the data, and the employed methods all influence the choice of data type. Models for machine learning and deep learning are trained using data that are most pertinent to the purpose for which they were created. We used image data types in this paper. For predicting malignant lesions or benign lesions, a microscopic image can be used [9,10]; either a real-time high-regulated image or hyperspectral mouth image can also be used [11], and even a computed tomographic (CT) image [12] or fluorescence image can create a good dataset [13]. The field of computer vision is gaining popularity with convolutional neural networks (CNNs) [9,10,11,12,13]. However, large-scale, reliable clinical data are required for employing deep learning in the early diagnosis of oral cancer. The researchers examined a machine [14,15] for classifying oral cancer and dental disease. The contributions of this work are as follows.

- We added extra patch tokens and distillation tokens to the DeIT method for performing the classification effectively and detecting the lesions by providing 98.73% correct information for malignant lesions.
- We modified the structure of VGG19 and MobileNet by changing the fully connected (FC) layer, and both models give 100% accuracy.
- We established a standard comparison table with similar works. Among them, our models outperform on the basis of accuracy.

For benign and malignant lesion detection using transfer learning approaches, we formulate the following hypotheses:

- Null Hypothesis ($H_{0})$: there are no significant differences between the performances of fine-tuned transfer learning models;
- Alternative Hypothesis ($H_{a}$): the performances of fine-tuned transfer learning models have statistically significant differences.

A comparison between the results available in the literature and the result proposed in this study is shown in Table 1. The results show that MobileNet and VGG19 have much potential for medical use. On the other hand, DeIT offers an option that might be better at generalization.

## 2. Literature Review

Many researchers proposed different methods for finding the type of oral lesions using machine learning, deep learning, or transfer learning based on images of the lesions. The authors examined deep learning models for classifying and detecting oral lesions based on the images of the MeMoSA project. MeMoSA (Mobile Mouth Screening Anywhere) is a mobile phone app for collecting oral lesion images developed by Cancer Research Malaysia. They used ResNet-101 for classification, and for object detection, R-CNN was used. Furthermore, the accuracy (F1-score) was 87.07% for the lesion class, and for referral, it was 78.30% [16]. In [17], the authors analyzed oral mucosa disorders and their diagnosis through digital imaging and compared TNM (Tumor–Node–Metastasis) staging with a neural network for 75 patients, achieving high accuracy: 100% for T1, 85.19% for T2, 84.21% for T3, and 94.12% for T4. In [23], a study utilized deep transfer learning algorithms, like ResNet50, MobileNetV2, VGG19, VGG16, and DenseNet, on oral cancer images from histopathologic and real-time datasets. The most effective results were obtained with DenseNet using hybrid optimization techniques, achieving accuracies of 92.41% for real-time, 95.41% for oral cancer, and 92.41% for non-cancerous histopathologic images. Marzouk et al. [24] introduced the AIDTL-OCCM (Artificial Intelligence with Deep Transfer Learning Oral Cancer Detection and Classification Model), which used AI and image processing for oral cancer detection. It employs fuzzy-based contrast enhancement and DenseNet-169 for feature extraction, along with the Chimp Optimization Algorithm (COA) and an autoencoder (AE) for detection and parameter optimization. The results show that AIDTL-OCCM outperforms other methods, achieving a maximum accuracy of 90.08% in the early detection of oral cancer. Nanditha et al. [18] used texture and fractal features to analyze 200 oral lesion images from Karnataka medical institutions. A back-propagation neural network achieved 95% accuracy in detecting malignant and benign lesions, showing promise for improving oral cancer diagnosis. Rahman et al. [25] used transfer learning with AlexNet on oral squamous cell carcinoma biopsy images, achieving impressive classification accuracy of 97.66% for training and 90.06% for testing. In [20], an automated system for early oral lesion diagnosis was created using an ensemble deep learning model (Resnet-50 and VGG-16). Trained on an augmented dataset, it outperforms other models with 96.2% accuracy, 98.14% sensitivity, and 94.23% specificity. In [21], the authors applied six deep convolutional neural network (DCNN) models with transfer learning to automate the screening for oral cavity cancer (OCC) using a small dataset of tongue lesion images. The models successfully differentiated between benign and precancerous lesions and identified various tongue lesion types with an average accuracy of 93%. The authors proposed machine learning models with an accuracy of 86% for classifying oral cancers based on electronic health record (EHR) data [22]. In the study [25], it was observed that the highest achieved classification accuracy was around 97.66%. Therefore, there are research opportunities to improve the accuracy of the classification of lesion images in regard to the distinction between benign and malignant kinds. The primary objective of this work is to improve the precision of the classification of lesion images by distinguishing between benign and malignant kinds.

The problems and questions dealt with in our work are as follows:

- The efficacy of fine-tuned TL methods: The purpose of this work is to determine how successfully fine-tuned transfer learning models can be adapted to the job of categorizing oral lesions based on picture data.
- Classification accuracy: This work focuses on the classification process’s correctness, especially in discriminating between benign and malignant oral lesions.
- Comparison to existing works: This study compares the performance of fine-tuned transfer learning approaches to classic or conventional methods for oral lesion categorization.

In our work, we proposed three deep transfer learning (DTL) models, DeIT, VGG19, and MobileNet. VGG19 and MobileNet perform the best with an accuracy of 100% among the models. Therefore, the deep transfer learning (TL) model is proposed here to detect oral lesions or oral cancer and to classify benign and malignant lesions.

## 3. Proposed Methodology

**Figure 1:**
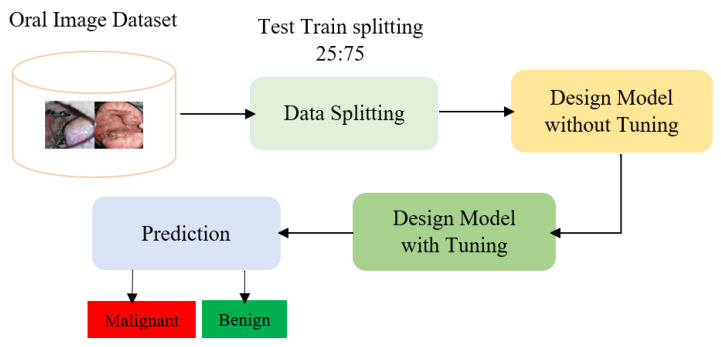
The proposed methodology.

Figure 1 deals with the block diagram of the methodology. First, the dataset is divided into training test and validation datasets, and then those data are preprocessed. Preprocessing is essential for achieving better accuracy. During splitting, the real data are used as a test dataset, the augmented data are used as a training dataset, and the same is used for validation. Then, we applied a deep transfer learning model for classifying the oral disease with fine-tuning models and not. The deep transfer learning model is a pre-trained model where knowledge of a model is applied to another model.

### 3.1. Dataset Description

The dataset is collected from the Mendeley platform [26], which contains color images of two kinds or types of oral lesions. The dataset was created using mobile cameras and intraoral cameras. After that, these images were consulted on with doctors from different hospitals in Karnataka, India. There were 165 benign lesions and 158 malignant lesions images in this dataset originally, and the augmentation technique was applied to increase this amount by up to 1320 training images for benign lesions and 1273 training images for malignant lesions. Three augmentation techniques named flipping, rotation, and resizing were used to extend the dataset.

### 3.2. Dataset Splitting and Enhancement

**Figure 2:**
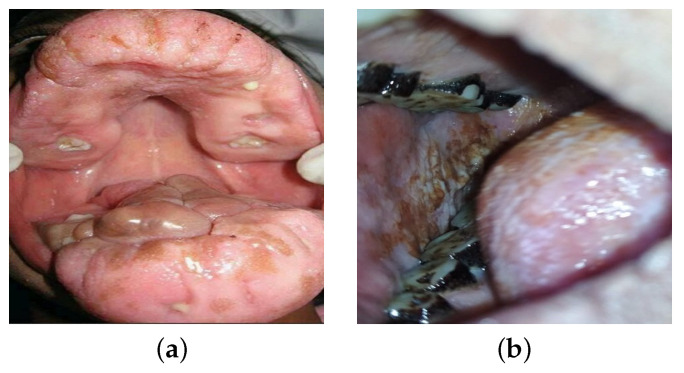
Oral lesion classes: (**a**) benign lesion; (**b**) malignant lesion.

The dataset contains 2593 images in total. Among them, 1320 images are benign lesions, and the rest 1273 are malignant lesions. This study collected 165 images of benign lesions and 158 of malignant lesions. Later, flipping, rotation, resizing, and cropping in word augmentation increased the data in the dataset. Figure 2 reflects the sample data from the dataset. We considered the image resizing, normalization, augmentation, color, and shape as features.

### 3.3. Transfer Learning Model Design

It is imperative at present in each sector of science, especially in medical science, to classify images depending on different features and properties. Transfer learning is becoming popular nowadays, as deep learning requires massive amounts of data and is time-consuming. Transfer learning gives opportunities to use the knowledge of previously used data. It helps to solve problems efficiently and reduces the demand for data. From a considerable dataset, many classes can be recognized using transfer learning. Even in computer vision problems, achieving expected accuracy using less data and computing resources is possible using the mentioned technique. Several transfer learning models are popular, like VGG16, ALexNet, ResNet, and so on [27,28,29]. Transfer learning or deep transfer learning (DTL) are related concepts using pre-trained models but have little difference. DTL is a more specialized transfer learning [30]. In this paper, we modified VGG19, MobileNet, and DeIT, which are well-performing transfer learning algorithms.

#### 3.3.1. VGG19 Model

**Figure 3:**
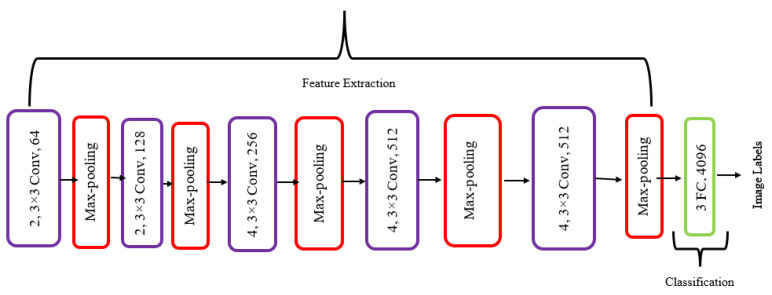
General VGG19 architecture.

VGG19 is a convolutional neural network model proposed in [31]. The model demonstrated a top-5 test accuracy of 92.7% on the ImageNet dataset, which included more than 14 million images dispersed across 1000 different classes. VGG19 is a variant of the VGG model, which uses 19 layers, including 16 convolutional layers, 5 max-pooling layers, and 3 fully connected layers. Figure 3 shows the regular architecture of the VGG19 model. VGG19 is widely used in applications such as object detection, image classification, and image segmentation [32].

Each convolutional layer uses a 3×3 filter and a ReLU (Rectified Linear Unit) activation function. Max-pooling layers use a 2×2 filter and a stride of 2. The input to the network is an image of size 224×224×3, where the first number is the height, the second is the width, and the third is the number of channels (RGB).

The convolutional layers apply a 3×3 filter to the image, which is then multiplied by a set of weights and passed through a ReLU function to produce an activation map. The max-pooling layers then combine the output of the convolutional layers into a single feature map, which is then fed into the fully connected layers. The fully connected layers consist of neurons with weights and biases that are used to classify the image.

**Figure 4:**
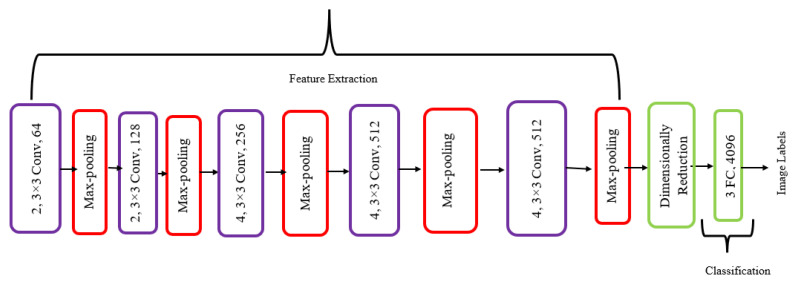
Modified VGG19 architecture.

In our architecture, we replaced the classification part with dimensional reduction and then added the classification, which is visually shown in Figure 4. Moreover, the hyper-tuning we have performed in the code is given in Table 2.

#### 3.3.2. MobileNet Model

**Figure 5:**
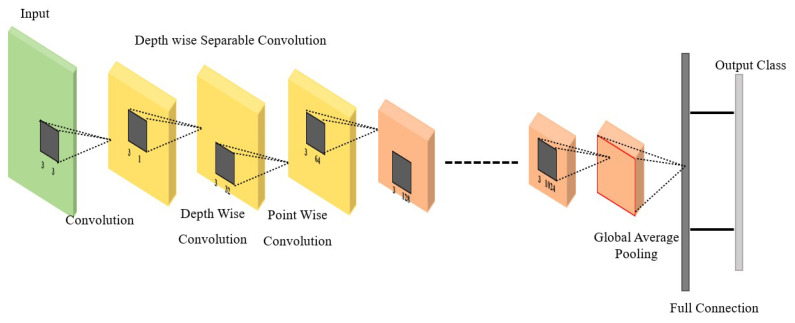
General MobileNet architecture.

MobileNet is a deep learning-based computer vision algorithm for efficient on-device image classification. Figure 5 shows that MobileNet combines depthwise separable convolutions and linear bottlenecks. The depthwise separable convolutions split the traditional convolution operation into two separate operations, reducing the number of computations and the number of parameters, which helps reduce the network’s size and computational complexity. The linear bottlenecks further reduce the number of parameters in the network and make it more computationally efficient [33].

In the case of MobileNet, the convolutional layers are replaced with depthwise separable convolutions. A depthwise separable convolution is a two-step operation that first applies a single filter to each input channel and then concatenates the results. This approach reduces the number of parameters and computations compared to a traditional convolution, making the network more efficient.

The linear bottlenecks in MobileNet refer to 1×1 convolutional layers added between the depthwise separable convolutions. These bottlenecks reduce the number of input channels, reducing the network’s computational and memory requirements. In the modified architecture, we used global max pooling instead of global average pooling. Our hyper-tuning for the model is shown in Table 3.

#### 3.3.3. DeIT Model

**Figure 6:**
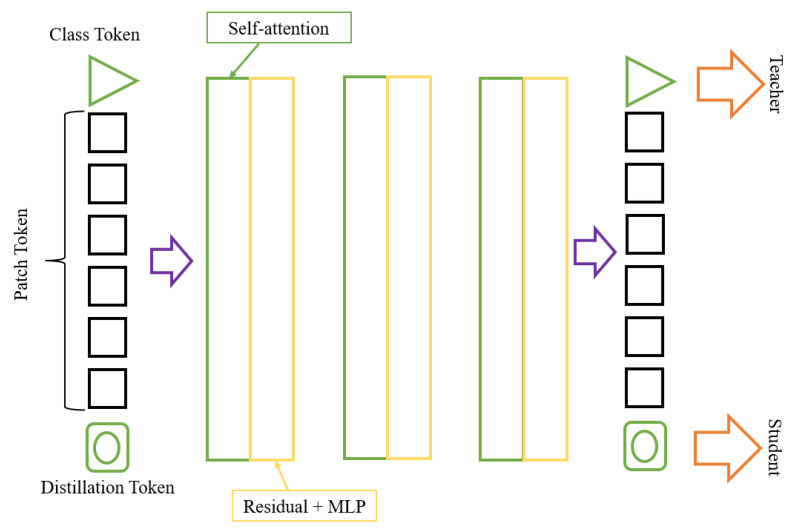
General DeIT architecture.

**Figure 7:**
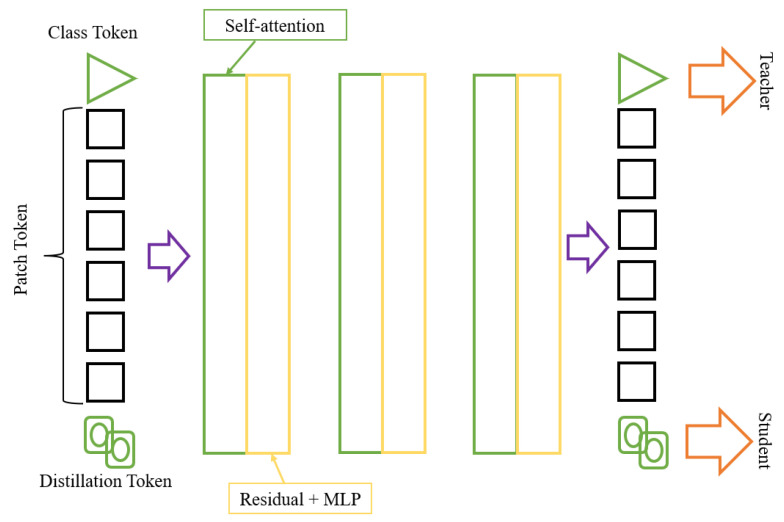
Modified DeIT architecture.

Image transformation techniques are used to improve the appearance of digital images. The improving techniques include improving the clarity, sharpness, contrast, brightness, and color. DeIT is a technique used to improve the quality of digital images automatically. The improving technique is performed using a deep learning algorithm to learn the features of a digital image and then apply appropriate transformations to improve the image. This technique can enhance various medical, industrial, artistic, and recreational images. DeIT can also automatically enhance the quality of images taken with mobile devices, making them look better without manual intervention [34]. The real architecture of this model is shown in Figure 6, and our modified architecture is shown in Figure 7. Table 4 shows the hyper-tuning for the model.

Some important terms regarding DeIT are the following:

- Interpolation: used to estimate the value of unknown points using known nearby points.
- Convolution: a mathematical operation that combines two functions to create a third function.
- Fourier transform: a mathematical tool representing a signal or image as a sum of sine and cosine waves of different frequencies.
- Resampling: the process of changing the resolution of an image by altering the number of pixels.
- Normalization: the process of adjusting the data values to a common scale.
- Histogram equalization: a contrast adjustment technique used to improve the contrast of an image by redistributing the pixel intensities.
- Edge detection: the process of locating sharp changes in an image.
- Affine transformation: a mathematical tool to transform an image by modifying its shape, size, and orientation.

### 3.4. Experimental Preparation and Assessment

Our dataset is a collection of substantial image sets. So, for training or testing purposes, a high-configuration machine is required. Jupyter Notebook is a way to run the program, as our dataset is split into training, testing, and validation before preprocessing. Our proposed model combines OpenCV, pickle, numpy, seaborn, matplotlib, Keras, TensorFlow, and sklearn.

**Figure 8:**
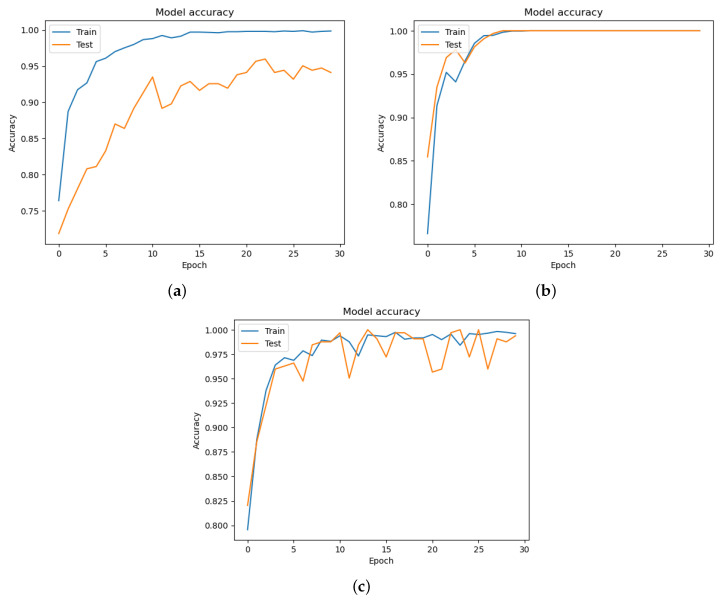
Accuracy of the models: (**a**) DeIT accuracy; (**b**) VGG19 accuracy; (**c**) MobileNet accuracy.

**Figure 9:**
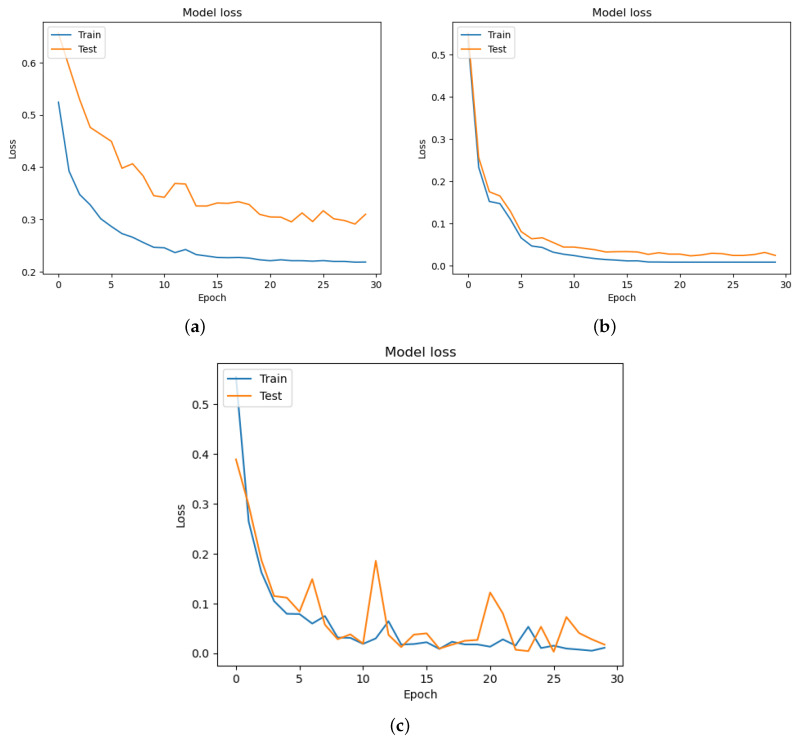
Loss function characteristics of the models: (**a**) DeIT; (**b**) VGG19; (**c**) MobileNet.

The cross-entropy loss is calculated among the training and valid datasets at each epoch. The cross-entropy was categorical for our proposed models. The Adam optimizer uses the learning rate of 0.1% [35]. All our models (DeIT, VGG19, ResNet) have been trained for 30 epochs. The accuracy of each model is described in Figure 8, where Figure 8a is of DeIT, Figure 8b is of VGG19, and Figure 8c is of MobileNet. The loss function of the proposed models is presented in Figure 9. The loss graphs of DeIT in Figure 9a, VGG19 in Figure 9b, and MobileNet in Figure 9c illustrate the points of the validation and training dataset.

## 4. Result Analysis and Discussion

We evaluated five performance metrics including the precision, recall, F1-score, MCC, and accuracy for all DTL models. All these performance parameters can be measured using the confusion matrix. There are four keywords in the confusion matrix named true positive (TP—it means when the actual value and the prediction value are positive), true negative (TN—it means when the actual value and the prediction value are negative), false positive (FP—it means when the actual value is negative but the prediction value is positive), and false negative (FN—it means when the actual value is positive but the prediction value is negative). All performance metrics are mathematically defined in Equations (1), (2),(3), (4), and (5), respectively.

Recall is the ratio of the total number of true positive classes to the predicted true positive class.

(1) $$Recall,R=\frac{TP}{TP+FN}$$

Precision is the ratio of correct positive guesses to total (correct and false) positive guesses.

(2) $$Precision,P=\frac{TP}{TP+FP}$$

The F1 measure is the harmonic average of precision and recall.

(3) $$F1Score,F1=2\times \frac{P\times R}{P+R}$$

Accuracy is the ratio between the number of correct predictions and the total number of predictions.

(4) $$Accuracy=\frac{Number\;of\;correct\;predictions}{Total\;number\;of\;predictions\;made}$$

The Matthews Correlation Coefficient (MCC), also known as the phi coefficient, is a measure used in machine learning and statistics to evaluate the quality of binary classification models, particularly when dealing with imbalanced datasets.

(5) $$MCC=\frac{TP\times TN-FP\times FN}{\sqrt{\left(TP+FP)\times (TP+FN)\times (TN+FP)\times (TN+FN\right)}}$$

The results of our study showed that all three models, DeIT, VGG19, and MobileNet, achieved high levels of accuracy in detecting oral lesions. Accuracy graphs for all the models are shown in Figure 8. In particular, the VGG19 and MobileNet models had a perfect accuracy rate of 100%, while the DeIT model achieved a slightly lower accuracy rate of 98.86%. From precision, recall, and F1-score metrics for each model, as shown in Table 5, we found that the DeIT model had a precision rate of 96.81%, recall rate of 96.68%, and F1-score of 96.68%. Meanwhile, the VGG19 and MobileNet models had perfect precision, recall, and F1-score values of 100%. We also calculated the Matthews Correlation Coefficient (MCC) for each model to evaluate its performance and found that the VGG19 and MobileNet models had perfect MCCs of 100%, while the DeIT model had an MCC of 93.49%.

These results suggest that all three models effectively detect oral lesions, with the VGG19 and MobileNet models achieving perfect accuracy rates. The DeIT model also performed well, with high precision, recall, and F1-score values and a relatively high MCC value.

**Figure 10:**
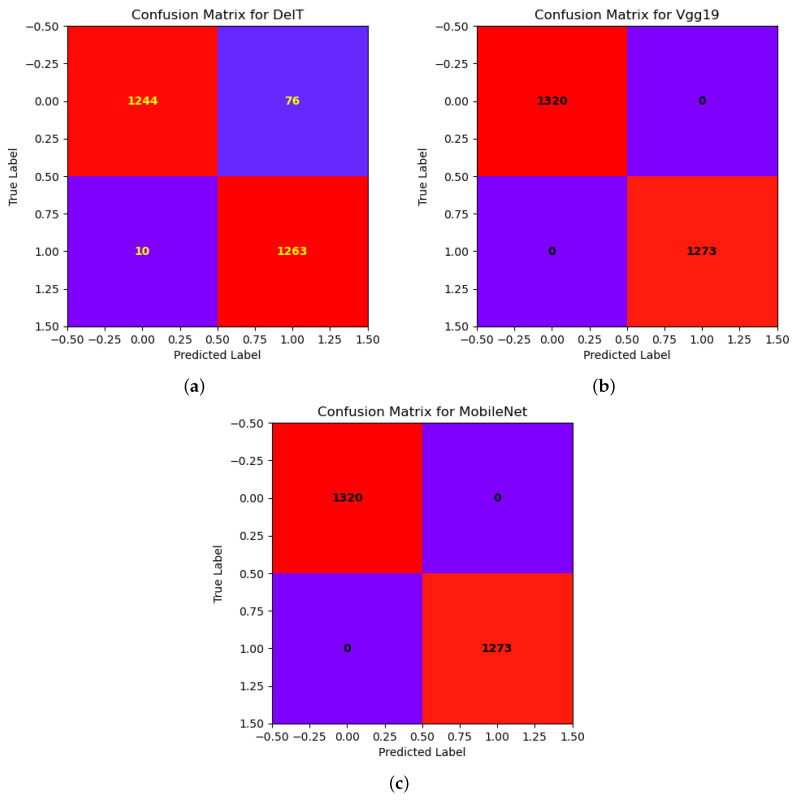
Confusion matrices of the models: (**a**) DeIT; (**b**) VGG19; (**c**) MobileNet.

We have shown the performance of each model through the use of loss, as shown in Figure 9, and accuracy graphs, as shown in Figure 8, which indicated that all three models were able to converge to a low loss and high accuracy after several epochs of training. Additionally, we created confusion matrices for each model to visualize their performances in identifying true positives, true negatives, false positives, and false negatives, as shown in Figure 10.

The confusion matrices for the models show that false negatives were more common than false positives, as shown in Figure 10. The figure shows that models are better at detecting benign/malignant lesions than correctly classifying them. The false negatives could be due to the difficulty distinguishing between benign and malignant lesions, especially for images with ambiguous features.

### 4.1. Cross-Validation

We applied stratified cross-validation techniques to evaluate the performance of the models. The results of all models using stratified cross-validation are shown in Table 6. This table demonstrates almost the same results as the one without validation.

### 4.2. Discussions

The results of our study indicate that deep learning models can effectively detect oral lesions with high accuracy. The 100% accuracy achieved by MobileNet and VGG19 highlights the potential of using these models in clinical settings. However, it should be noted that DeIT achieved a high accuracy of 98.73%, which is still very promising for practical use. The superior performances of MobileNet and VGG19 can be attributed to their deeper architectures and the use of pre-trained weights. These models could learn and distinguish between different lesion features with high accuracy, even with a relatively small dataset. Due to its transformer-based architecture, DeIT may have a greater capacity to generalize to unseen data despite showing slightly less accuracy. The utilized models have some drawbacks. They are as follows:

- VGG19 has some significant drawbacks. These include its high memory requirements, inefficiency for installations with limited resources, and high computational complexity. It is made just for picture data, frequently has to be adjusted, and is ineffective in capturing spatial hierarchies. Furthermore, the quality and quantity of training data have a significant impact on its performance, which raises issues with fairness when biased data are present.
- Despite its effectiveness, MobileNet is not without its restrictions. It is not well adapted for tasks like natural language processing or textual data. It may struggle with fine detail detection, be inaccurate in complicated picture recognition, and perform best in generic image categorization. Its small size could make it less capable of handling high resolutions or bigger photos. Finally, understanding its decision-making process might be difficult.

We have some direction for the future research’s scope. The first is dataset scarcity. There are only 165 benign lesions and 158 malignant lesions. This could be increased in the future. The second is to experiment with more DTL models, such as the three models applied to classify oral lesions in this paper. It could be a combination of two or more models.

We covered all the mentioned questioning problems in this paper. We did fine-tune all the models. Table 2, Table 3 and Table 4 show the hyper-parameters of all models for fine-tuning. The accuracy of all models is shown in Table 5. Table 1 shows the comparison with existing works.

## 5. Conclusions

This study showed the efficiency of deep learning models in identifying benign and malignant oral lesions. The comparative analysis among the three techniques—DeIT, VGG19, and MobileNet models—showed promising capabilities for detecting oral lesions. MobileNet and VGG19 have much potential for medical use, as the experiment results showed they had the highest accuracies. However, DeIT offers an option that might be better at generalization. This study also showed the importance of using “data augmentation” methods to make the datasets bigger and make the models work better. In addition, the results show that loss and accuracy graphs and confusion matrices are performance-indicating parameters for oral lesion classifications. Several metrics were used to evaluate how well the models worked, including their precision, recall, F1-score, MCC, and accuracy. The evaluation metrics show that all three models performed well in classifying oral lesions. Significantly, the VGG19 technique achieved perfect performance parameters—its accuracy, precision, recall, F1-score, and MCC. The dataset included in this research is of a limited size (total images). It comprises images of patients from a specific geographic region inside the area. Hence, it is imperative for the approach to undergo further examination using a diverse set of lesion images. For future work, this study recommends further exploring more advanced data augmentation techniques and deep learning architectures to improve the models’ performance. Additionally, this study suggests exploring transfer learning techniques to fine-tune pre-trained models on a specific oral lesion dataset. Furthermore, this study suggests assessing the effectiveness of the models on large datasets that cover diverse geographical regions to ascertain their effectiveness.

## Figures and Tables

**Table 1:** Comparison among the existing works.

Reference	Year	Model	Accuracy
Welikala et al. [16]	2020	ResNet-101	87.07%
Prabhakar and Rajaguru [17]	2017	TNM	90.88%
Nanditha et al. [18]	2020	BPNN	95%
Thomas et al. [19]	2013	ANN11	97.92%
Nanditha [20]	2021	VGG16	96.2%
Shamim et al. [21]	2019	VGG19	96.7%
Wu et al. [22]	2019	AlexNet	91%
Proposed	2023	VGG19 and MobileNet	100%

**Table 2:** Hyper-tuning in VGG19.

Parameter	Parameter Value
Batch size	32
Weight matrix	imagenet
Activation function	softmax
Criterion	sparse_categorical_crossentropy
Optimizer	Adam

**Table 3:** Hyper-tuning in MobileNet.

Parameter	Parameter Value
Batch size	32
Weight matrix	Imagenet
Activation function (dense: 1024)	ReLU
Activation function (dense: 512)	ReLU
Activation function (dense: 2)	softmax
Criterion	categorical_crossentropy
Optimizer	Adam
Learning Rate	0.02

**Table 4:** Hyper-tuning in DeIT.

Parameter	Parameter Value
Batch size	128
Weight matrix	Linear
Activation function	ReLU
Regularization	Dropout
Criterion	LabelSmoothingCrossEntropy
Optimizer	Adam
Learning rate	0.001

**Table 5:** Summary of the result where P = precision and R = recall.

Model	P (%)	R (%)	F1 (%)	MCC (%)	Accuracy (%)
DeIT	98.73	94.24	96.38	93.49	96.40
VGG19	100	100	100	100	100
MobileNet	100	100	100	100	100

**Table 6:** Performance analysis of all models using cross-validation where P = precision and R = recall.

Model	P (%)	R (%)	F1 (%)	MCC (%)	Accuracy (%)
DeIT	98.75	94.5	96.54	93.58	96.70
VGG19	100	100	100	100	100
MobileNet	100	100	100	100	100

## Data Availability

The datasets utilized in this article were obtained from the Mendeley Data webpage, which is freely accessible for all scientists and investigators to conduct experiments and can be accessed at the following website: https://data.mendeley.com/datasets/mhjyrn35p4/2, (accessed on 7 August 2023).
